# Editorial: The interconnection between the tumor microenvironment and immunotherapy in brain tumors

**DOI:** 10.3389/fonc.2023.1147883

**Published:** 2023-03-13

**Authors:** Chunrun Qu, Longbo Zhang, Wen Cheng, Quan Cheng, Junxia Zhang

**Affiliations:** ^1^ Department of Neurosurgery, Xiangya Hospital, Central South University, Changsha, Hunan, China; ^2^ Xiangya School of Medicine, Central South University, Changsha, Hunan, China; ^3^ National Clinical Research Center for Geriatric Disorders, Xiangya Hospital, Central South University, Changsha, Hunan, China; ^4^ Department of Neurosurgery, Yale University School of Medicine, New Haven, CT, United States; ^5^ Department of Cellular & Molecular Physiology, Yale University School of Medicine, New Haven, CT, United States; ^6^ Department of Neurosurgery, Shengjing Hospital of China Medical University, Shenyang, China; ^7^ Department of Neurosurgery, The First Affiliated Hospital of Nanjing Medical University, Nanjing, Jiangsu, China

**Keywords:** immunity, brain tumor, tumor microenevironment, immunity checkpoint, tumor infiltrating cell

The tumor microenvironment (TME) is an essential part of cancer and plays a key role in regulating tumor growth, progression, migration, and response to therapy in several types of cancer, including brain tumors. The TME is a complex network of cancer cells, stromal cells, and tumor-infiltrating immune cells (TIICs), in which tumor cells communicate not only with each other but also with stromal cells and immune cells. A deep understanding of the interactions between tumour cells and the TME will help us to predict patient prognosis, reduce drug resistance and develop new therapies. Immunotherapy, represented by immune checkpoint (ICP) blockade and chimeric antigen receptor (CAR)-T-cell therapy, has revolutionized cancer treatment (1, 2). However, few clinical trials have demonstrated the beneficial effects of immunotherapy on survival in brain tumor patients.

Gliomas are the most common malignant primary brain tumours in adults and are characterized by recurrence, poor prognosis, and treatment resistance. The main reason for the poor prognosis is the high heterogeneity of tumor cells and their immunosuppressive TME. TIICs are currently thought to be abundant in the immunosuppressive TME of brain tumours and are associated with prognosis. Several key roles for TIICs in brain tumours have also been reported. Current conventional therapies have failed to improve the clinical prognosis, and innovative therapeutic approaches are needed.

The Research Topic consists of 26 manuscripts focusing on various aspects related to “The Interconnection Between the Tumor Microenvironment and Immunotherapy in Brain Tumors”. We have summarized what is of particular interest in these manuscripts, including the following sections: (i) TIIcs in the TME; (ii) the relationship between ICPs and immunotherapy; and (iii) modification of RNA.

## TIICs in TME

TIICs play an important role in promoting or inhibiting tumour growth. The study of TIICs has been instrumental in improving the understanding of the TME in glioma and improving the effectiveness of immunotherapy.

Macrophages are an important population of immune cells in the TME of gliomas that have immune functions and can be involved in tumour development through the release of a variety of cytokines and growth factors. Activated macrophages are usually divided into M1 macrophages, which have a predominantly antitumour response, and M2 macrophages, which promote tumour cell development and metastasis. Zhou et al. found that M2 macrophages were abundant in malignant gliomas and were associated with poor prognosis. PTEN is the most often lost tumour suppressor in primary gliomas. They also found that high PTEN expression was associated with prolonged survival in glioma patients and polarization of macrophages. This suggests that the detection of changes in PTEN status will play an important role in predicting a patient’s response to immunotherapy.

Autophagy is a key regulatory point for the interaction between glioma and the TME. Autophagy enhances the drug resistance and invasiveness of glioma cells and differentiates major macrophages to the M2 phenotype, inhibiting their phagocytosis and promoting glioma progression. Recent findings on glioma and the TME presented by Fan et al. from the perspective of autophagy may enhance the understanding of the TME and stimulate more applicable and effective strategies for targeting the TME while using autophagy to synergistically fight cancer.

Although specific markers of T cell exhaustion and activation have been studied in glioblastoma (GBM), the migration receptors they require in homing remain unknown. The expression profiles of integrin and chemokine receptors in endogenous GBM-infiltrating T cells were described and validated by Kollis et al. through single-cell RNA sequencing. This has very positive implications for improving the efficacy of T-cell immunotherapy.

Natural killer (NK) cells are a possible next research hotspot. Pan et al. summarized the characteristics of NK cell therapy in glioma treatment. The low incidence of cytokine release syndrome in patients with CAR-NK therapy significantly compensates for the disadvantages of CAR-T cell therapy. CAR-NK cells cause very little graft-versus-host disease. In addition, CAR-NK cells can kill their targets in a CAR-independent manner. They also summarized ways in which the effectiveness of the treatment may be further improved.

MXRA8 is thought to be associated with iron death and is a novel prognostic indicator. Xu et al. found that MXRA8 was significantly associated with various infiltrating immune cells, and played a key role in ferroptosis and the TME in gliomas, promoting tumour progression.

## ICPs

Evolving tumour immune evasion mechanisms play an important role in the development and progression of tumours. One of the main mechanisms is related to the immune checkpoint pathway. Immune checkpoint molecules regulate the immune system and maintain its stability by stimulating and suppressing immune responses in physiological situations, but they may also be involved in cancer development. Programmed death protein 1 (PD-1) and cytotoxic T lymphocyte-associated protein 4 are two extremely important immune checkpoint proteins.

PD-1 is expressed on many immune cells and interacts with its ligands, programmed death-ligand (PD-L)1 and PD-L2, upon binding to suppress immune responses. The antitumour immune responses mediated by CD8+ T cells are suppressed by PD-L1. The tumor-infiltrating CD8+ T cell/PD-L1 axis is significant for GBM therapy, but its exact role is not clear. According to a meta-analysis by Shadbad et al., patients with untreated GBM who have PD-L1 overexpression may have a poor prognosis. Increased tumour-infiltrating CD8+ T cells in tumour tissue of patients receiving anticancer therapy may be linked to a worse prognosis because radiotherapy/chemotherapy can upregulate PD-1 expression in tumour-infiltrating CD8+ T cells as well as induce immune cells in a state of exhaustion. lncRNAs are thought to play a critical role in regulating tumour immunity, influencing the activation and function of T cells and macrophages. Polymerase 1 and transcript release factor (PTRF) expression is increased in mesenchymal phenotype GBM (3). According to Yi et al., PTRF interacts with the long noncoding RNA (lncRNA) NEAT1 to stabilize its mRNA, and through NEAT1, PTRF inhibits the expression of UBXN1, which in turn enhances NF-κB activity and, through this pathway, PD-L1 expression. Ultimately, PTRF promotes GBM immune evasion by affecting PD1-PD-L1 interactions. The PTRF-NEAT1-PD-L1 axis may be a novel immunotherapeutic target in GBM. In various cancer types, the lncRNA HOTAIR is highly expressed and linked to tumor metastasis. HOTAIR expression is also elevated in the mesenchymal phenotype of GBM (4). Wang et al. reported that HOTAIR was an activator of the NF-κB pathway lncRNA, its levels positively correlated with PD-L1 levels, and its apparent upregulation promoted inflammatory signaling and immune escape in glioma cells.

PD-L2 is also a PD-1 ligand that can mediate cancer cell immune escape. Xie et al. showed that PD-L2 was overexpressed in glioma tissue and was associated with poor prognosis and immune cell infiltration. In addition, PD-L2 may be a promising predictive biomarker for selecting the best individualized treatment strategy.

## Modification of RNA

The study of RNA epitranscriptomics is at the forefront of the field of specific chemical modifications of biomolecules. RNA can have multiple chemical modifications that affect gene expression to varying degrees. Their importance in cancer is gradually being discovered, opening up new possibilities for cancer therapy. Important internal RNA modifications include 5-methylcytosine (m5C), N6-methyladenosine (m6A), 7-methylguanosine, N1-methyladenosine and others (5).

The most widespread internal modification of RNA is the methylation of adenosine at the 6-position to give m6A. Proteins involved in this process are m6A “writers”, m6A “erasers” and m6A “readers”. The “writers” are responsible for the installation of m6A, including METTL3 and METTL14, among others (6). The “erasers” have demethylation capabilities and are responsible for the deletion of m6A, including ALKBH5 and FTO, among others (7, 8). “Readers” regulate m6A expression, including YTHDF1 and YTHDF2 (9, 10). m6A modification has been shown to affect the progression of many cancers; for example, in breast cancer, METTL3, HBXIP and let-7g miRNAs form a positive feedback loop to promote cell proliferation (11). However, the relationship between m6A modifications and the TME in GBM is not fully understood at present. The relationship between m6A regulators and other factors was examined by Yuan et al. using bioinformatics and statistical methods. They discovered that at the single-cell level, m6A alterations promote GBM cancer cells to be in a stem cell state and, through ICPs and the GALECTIN signaling pathway, support the development of an immunosuppressive TME. They also identified two m6A-ICP expression patterns (high m6A/low ICP and low m6A/high ICP). ALKBH5 is a m6A “eraser” located in the nucleus, and overexpression of ALKBH5 results in low levels of m6A. Gene Ontology and Kyoto Encyclopedia of Genes and Genomes enrichment analysis by Wei et al. showed that ALKBH5 is associated with inflammatory, metabolic and immune processes in gliomas. ALKBH5 expression also affects sensitivity to immunotherapy and is associated with ICP expression. The ALKB family in GBM underwent thorough analysis by Feng et al. They found that the expression levels of ALKBH2 and ALKBH8 were significantly increased in GBM compared to normal tissues and that greater expression was associated with higher levels of mRNA processing and poorer patient prognosis. This study also showed a close link between DNA damage repair in GBM and the biological activity of members of the ALKB family.

Based on 32 regulatory genes, Xu et al. comprehensively assessed 539 non-m6A RNA modification patterns in GBM and connected these changes to TME invasion and mesenchymal conversion traits. They ultimately identified three patterns of non-m6A RNA modifications associated with different clinical features and biological pathways. This investigation uncovered possible connections between non-m6A RNA modification regulators and clinical characteristics, TME status, and GBM subtypes and clarified their therapeutic potential.

Although the prognosis for patients with lower-grade glioma (LGG) is relatively good, patients frequently relapse and tend to progress to higher grade gliomas, ultimately leading to treatment failure. Eight lncRNA m5C-associated prognostic signatures were established and validated by Zhou et al. for predicting prognosis and treatment response in LGG patients. This provides a promising target for further research.

## Prospective

**Figure 1:**
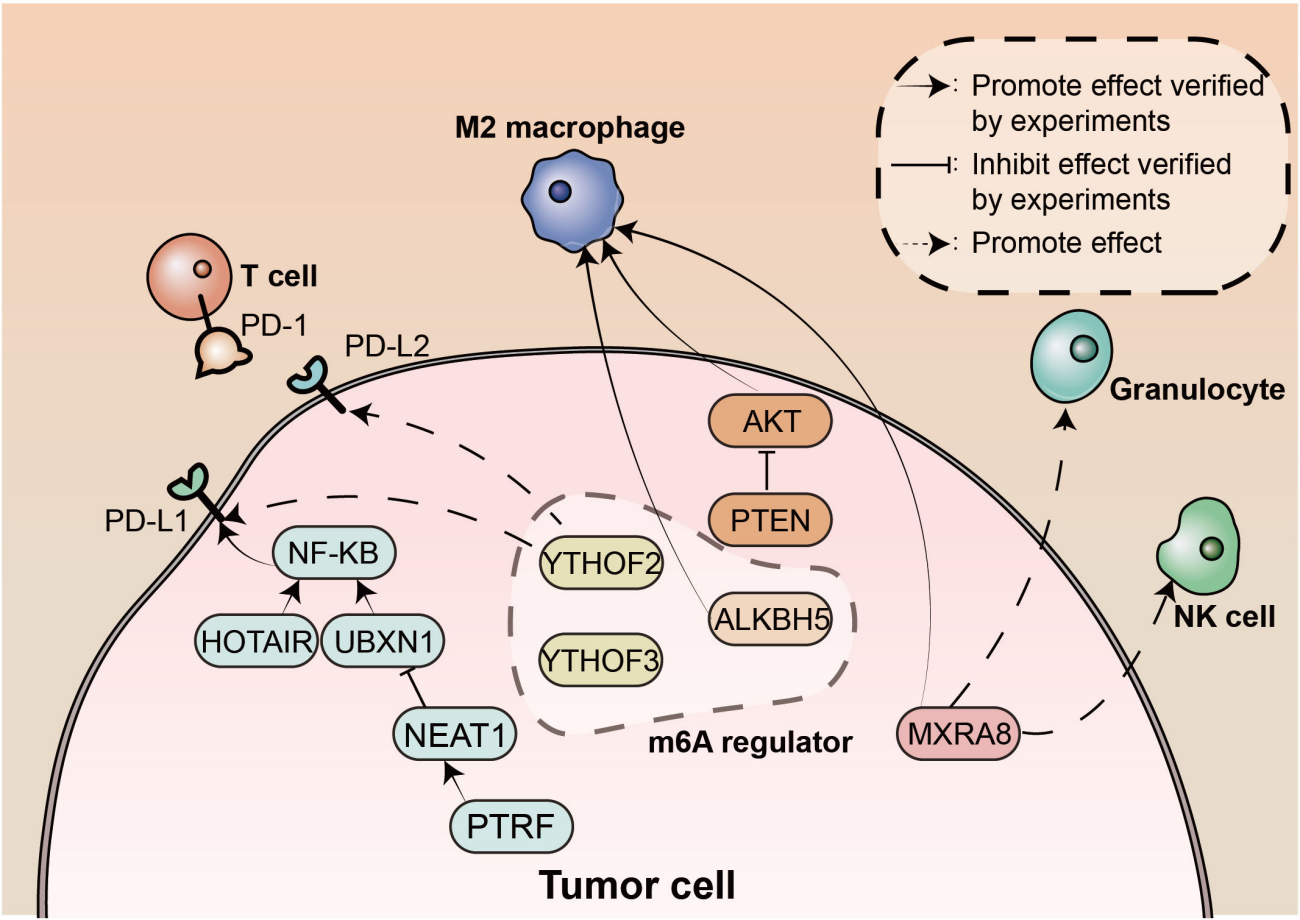
The landscape of tumor microenvironment in brain tumors. m6A, N6-methyladenosine; NK, natural killer; PD-1, Programmed death protein 1; PD-L1, programmed death-ligand 1; PD-L2, programmed death-ligand 2; PTRF, polymerase 1 and transcript release factor.

This editorial summarizes the key findings of the Research Topic “*The Interconnection Between the Tumor Microenvironment and Immunotherapy in Brain Tumors*”. Here, we present an understanding of the interactions between TIICs in the TME of brain tumours, the potential prognostic factors in the TME that predict patient survival, and the impact of specific components of the TME on the efficacy of immunotherapy or other oncological treatments. Important findings are summarised in **Figure 1**. Immunotherapy has shown great potential in a variety of cancers, and we hope that this Research Topic will contribute to an increased awareness and understanding of the link between the tumour microenvironment and immunotherapy in brain tumours, providing an opportunity to improve treatments and improve patient prognosis.

## Author contributions

All authors listed have made a substantial, direct, and intellectual contribution to the work and approved it for publication.
